# A Study Protocol for Persons With Neurological Diseases: Linking Rehabilitation Goals to the International Classification of Functioning, Disability and Health With a Focus on Assistive Technology for Cognition and Its Effects

**DOI:** 10.3389/fresc.2022.864804

**Published:** 2022-05-10

**Authors:** Helen Lindner, Nina Buer

**Affiliations:** ^1^School of Health Sciences, Örebro University, Örebro, Sweden; ^2^Center for Adult Rehabilitation, Faculty of Medicine and Health, Örebro University, Örebro, Sweden

**Keywords:** assistive technology for cognition, neurological disease, goal, community rehabilitation, longitudinal effect

## Abstract

Persons with neurological diseases often have some degree of cognitive impairment. They are in need of assistive technology for cognition (ATC) to compensate for cognitive impairments that affect their daily functioning. Goal setting in relation to cognitive deficits using ATC are common in clinical practice, and therapists often set several rehabilitation goals together with a patient. However, these rehabilitation goals are usually phrased differently, which limit the comparison of ATC and rehabilitation goals. It is thus valuable to link the goals to some standardized terminologies, such as the International Classification of Functioning, Disability and Health (ICF). Furthermore, goal achievement is seldom used to evaluate long-term effects of ATC in persons with neurological diseases and limited attention has been paid to the factors that predict goal achievement in using the ATC as cognitive support in persons with neurological diseases. The aim of the project is 3-fold. Firstly, we will use the ICF to link rehabilitation goals regarding the use of ATC in adults with neurological diseases. Secondly, we will evaluate effects of the ATC using goal achievement over a 5-year period. Thirdly, we will explore the variables that predict goal achievement in relation to the effects of ATC.

## Introduction

Neurological diseases are diseases of the central and peripheral nervous systems that negatively affect cognition, independence, and daily functioning. They are one of the leading causes of disability and unfortunately, advances in prevention and management of major neurological disorders are not sufficiently effective to encounter global burden of neurological diseases (1). Persons with neurological diseases often have some degree of cognitive impairment, which include attention deficits, visual perception deficits, sensory processing deficits, slow processing speeds, short-/long- term memory difficulties, and executive dysfunctioning (2, 3). They are often in need of assistive technology for cognition (ATC) to compensate for cognitive impairments that affect their daily functioning.

ATC are technologies designed to compensate for cognitive impairments as we mentioned earlier. Most of the ATC available in the commercial market are designed for the areas of time/day orientation, organization of daily activities and management of daily routine. Common examples of ATC are visual and audio reminders to perform a given task at a particular time, electronic clocks that visualize the time in different colors or modes (analog or digital), electronic calendars that visualize days and weeks and electronic planners that visualize activities during the day and week (4, 5). The goals of ATC are to compensate for cognitive impairments, increase independence and reduce the need for personal assistance (6, 7). ATC can thus be seen as cognitive support for “someone” (a person with cognitive impairment) doing something (an activity) somewhere (in a particular environment) (4). A number of reviews have reported the benefits of ATC in adults with neurological diseases (5–8). The main findings were that ATC effectively provides support for cognitive functions relating to attention, calculation, emotion, experience of self, higher level cognitive functions (planning and time management) and memory. Smartphones, personal digital assistants (PDAs), etc. with e.g., calendars and reminder alarms can improve prospective memory (8).

However, it is a complex interaction among the person with neurological disease, the activities that she or he wants to engage in, the environment or context in which the activity occurs, and the ATC that is available to support the activity. The International Classification of Functioning, Disability and Health (ICF) (9) describes human functioning and disability in relation to contextual factors; and has the potential to accommodate this complex interaction. The ICF organizes human functioning into two parts. Part 1 has two components: body functions and body structure, activity and participation. Part 2 also has two components: environmental factors and contextual factors. Each component consists of a number of categories with clear definitions. The ICF team and experts in neurological conditions have developed two ICF core sets for neurological conditions, one for the acute phase and the other for the post-acute rehabilitation phase (10, 11), however, these two core sets may not be fully applicable for patients with neurological diseases in the community rehabilitation phase. During the community rehabilitation phase, the conditions of patients with neurological diseases are relatively stable; some of the patients may have the opportunity to return to work part-time or establish a better daily routine, and most importantly, they would like to be as independent as possible.

During the community rehabilitation phase, patients with neurological diseases need cognitive support (ATC) to be able to perform different activities, alone or with others in different environments. Often, goal setting in this rehabilitation phase is important because it motivates both the patient and the rehabilitation team to work toward the same goal (12). Goal setting in relation to cognitive deficits using ATC are common in clinical practice and therapists often set several rehabilitation goals together with a patient. However, these rehabilitation goals are usually phrased differently, which limits the comparisons of rehabilitation goals in relation to the ATC. Particularly at the level of participation, goals are very much dependent on the person's daily habits and occupation. It is thus valuable to link the goals to some standardized terminologies, such as the ICF. Linking rehabilitation goals to the ICF will provide a comprehensive overview of the goals in relation to the use of ATC as cognitive support and help to identify the aspects that are lacking in rehabilitation service for persons with neurological diseases. One question is whether the ATC available in the market only support certain cognitive functions or a broad range of cognitive functions. Linking the rehabilitation goals to the ICF will provide us a good answer to this question.

However, although goal setting in relation to the prescription of ATC is a common clinical practice, effects of ATC in relation to goal achievement are seldom investigated in persons with neurological diseases. Measuring goal achievement in relation to the effects of ATC is to measure the extent to which patient's individual goals are achieved after the prescription of ATC. Particularly, there is a lack of literature that reports goal achievement in relation to the use of ATC to support different levels of participation, such as cognitive support for returning to work, or cognitive support for social functioning. Furthermore, despite the potential benefits of ATC reported in numerous studies, there are very few longitudinal studies that report the long-term effects of ATC in persons with neurological diseases. Long-term effects are crucial for different reasons. First, a long-term follow-up evaluation will provide a better picture whether the technology has been fully integrated into the user's everyday life. Second, learning and using ATC often requires a substantial degree of cognitive function, especially for those with moderate cognitive impairments. A long-term follow up will show the learning progress of ATC users. Third, development of ATC is advancing rapidly, and new ATC are released every year. Long-term follow up of the usability of ATC will show a better view of any improvement in human-machine interface. Fourth, the aim of ATC is to increase independence of the patients and reduce the burden on caregivers. However, environmental factors, such as caregivers that are close to the person, can be either barriers or facilitators in the use of ATC (13). It is important to identify the factors that would predict goal achievement in using the ATC as cognitive support in persons with neurological diseases. Conclusions that will be drawn from a long-term follow up of ATC will influence our services, prescription, policymaking, and the expenditure of public funds.

Thus, the aim of the study is 3-fold. Firstly, we will use the ICF to link rehabilitation goals regarding the use of ATC in adults with neurological diseases. Secondly, we will evaluate effects of the ATC using goal achievement over a 5-year period. Thirdly, we will explore the factors that predict goal achievement in relation to the effects of ATC.

## Method

### Study Design

A longitudinal prospective study will be used to answer the aim. The study is a 5-year project and data will be collected at different time points during a 5-year period.

### Participants

Inclusion criteria are (i) patients who have a neurological disease, (ii) patients who have a need for at least one ATC, and (iii) age 18 or over. We will exclude patients with neurological diseases who have other non-neurological diseases.

### Ethics

The study has received ethical approval from Swedish Ethical Review Authority. The number is Dnr 2021–01337.

### Recruitment

We will recruit patients with different neurological diseases *via* Adult Rehabilitation Center, Region Örebro County, Sweden. Power analysis based on multiple regression model suggests a total of 100 participants will be enough for data analysis. With a consideration of 40% dropout over the 5-year period, we plan to include 140 participants.

Information about the study will be sent to the homes of potential participants. The patients and their families will have the opportunity to ask questions about the study and how data will be extracted from their records. The patients will be assured that their refusal of participation will not affect the health care service they will continue to receive from the rehabilitation center. Once a patient has consented to take part in the study, a research assistant will start data collection from the patient's records.

### Data Collection and Instruments

We will extract data before, during and after the prescription of the ATC. The following data will be extracted:

1. Number of diagnoses and symptoms: the number of health conditions will be measured by summing the number of neurological diagnoses (according to the ICD-10 SE) and the number of neurological symptoms. For example, a patient can have two neurological diagnoses, such as stroke and epilepsy.
2. Demographic data: We will collect data on age, sex (0 = male, 1 = female), marital status (0 = single, 1 = married, 2 = divorced, 3 = widow), years of education, living arrangement (0 = living alone, 1 = not living alone), availability of caregiver (0 = no caregiver, 1 = have caregiver).
3. Performance in everyday activities: We will collect results of the performance in everyday activities using the ADL Taxonomy (14). It is an instrument that consists of 16 activities with three to six actions in each activity and it is routinely used by occupational therapists at the center. As for this study, the result of performance will be summed by the number of successful performances of each action (0 = cannot, 1 = can). The actions in the ADL Taxonomy are already mapped by its developers to the following equivalent ICF categories (Table 1) (14).
4. Assessment of cognitive functions during everyday activities: the Cognitive Checklist is a checklist that assesses 11 cognitive areas in one of the everyday activities in the ADL-Taxonomy (15). It is routinely used by occupational therapists at the center to assess cognitive functions in an everyday activity performed by patients. The results of the assessment will be summed by the number of successful areas (0 = cannot, 1 = can). The checklist assesses 11 cognitive areas and we have mapped the areas to the equivalent ICF categories (Table 2).
5. Choice of ATC: We will collect data on the ATC that will be chosen. The ATC will be primarily classified using the ICF cognitive functions (9) and according to Gillespie (16):
  - Attention Functions (b140)
  - Calculation Functions (b172)
  - Emotional Functions (b152)
  - Higher-level Cognitive Functions (b164)
  - Memory Functions (b144)
  Other ICF cognitive functions will be used to classify the ATC if none of the above ICF cognitive functions are appropriate. We will also classify the ATC using ICF categories in the environmental factor, e1151 Assistive products and technology for personal use in daily living.
6. Goal achievement using Goal Attainment Scale (GAS) (17): The GAS is an instrument that is intended to evaluate the effect of an intervention by assessing changes in a person's rehabilitation goals. The scale has five levels:
  −2: no change or current status
  −1: less than expected
  - 0: expected
  - +1: more than expected
  - +2: much more than expected

**Table 1:** Actions, activities and equivalent ICF categories in the ADL-Taxonomy.

**Activities**	**Actions and equivalent ICF category codes**
Reading and writing	• Writing signature, d170 • Writing messages, d345 • Reading mail/messages,d166 • Using own notes, d166 • Reading papers/books, d166 • Filling in forms,d166, d170
Handling communication aids	• Managing computer/tablet, d3601 • Managing phone/cell phone, d3600 • Managing TV/radio, d3608 • Answering phone calls, d3600 • Making phone calls,d3600
Orientation and Mobility	• In known indoor environment, d4600 • In known outdoor environment, d4602 • In unknown environment, d4602
Transportation	• Going by car, d4701 • Going by bus/tram/tube, d4702 • Going by train/boat/airplane, d4702 • Riding bicycle/moped, d4750, d4751 • Driving car/motorcycle, d4751
Personal hygiene	• Washing oneself, d510 • Bathing/shower, d5101 • Washing hair, d5100
Grooming and brushing teeth	• Shaving/make-up, d5200 • Manicuring, d5203 • Brushing teeth, d5201 • Combing one's hair/fix the hairstyle, d5202 • Pedicuring, d5204
Toileting	• Cleaning oneself after elimination, d5300, d5301 • Perform urinary and bowel elimination, d5300, d5301 • Arranging clothes, d5300, d5301 • Washing hands, d5100
Dressing	• Undress, d5401 • Dress, d5400 • Choose appropriate clothing, d5403 • Change to clean clothing, d5404 • Changing clothes in public spaces, d2401, d5400-5403
Eating and drinking	• Drinking alone, d560, d2102 • Eating and drinking together with others, d550, d560 • Eating alone, d550, d2102
Shopping	• Daily or small quantity shopping in neighborhood shop, d6200 • Making plans for shopping, d6200 • Weekly or large quantity shopping, d6200 • Shopping online, d6200, d3600
Cooking	• Heating up liquid or prepared food, d6300 • Preparing a cold meal, d6300 • Washing the dishes and pick up, d6300 • Cooking a hot meal, d6300
Cleaning	• Identifying need for cleaning, b1641 • Sorting wasted, 6405 • Daily light cleaning, d6401, d6403 • Weekly heavy cleaning, d6402, d6403 • Cleaning out, d6405
Washing	• Washing, d6400, d6403 • Planning for washing,d1641 • Taking care of clean laundry, d640
Handling economy	• Withdraw money, d860 • Paying bills, d865 • Having control over own economy, d870
Interactions with others	• Interacting with few people, d720 • Taking formal contacts, d740 • Interacting with many people, d720 • Ending contacts/relations, d7201 • Creating and maintaining intimate relationships, d770
Managing the day/time	• Getting up in time, d230 • Doing things in time/being on time, d220, d2303 • Getting to bed in time, d230 • Estimating time consumption, d2303

**Table 2:** The areas in the cognition checklist and the equivalent ICF categories.

**The areas in the cognitive checklist**	**The equivalent ICF categories**
Alertness and attention	b110 consciousness functions b140 attention Functions
Registration and recognition	b156 perceptual functions
Interpretation and gathering information	b117 intellectual functions
Memory	b144 memory functions
Time awareness	b1140 orientation to time
Initiating an activity	d2100 undertaking a simple task
Performing an activity with appropriate process skills	b164 higher-level cognitive functions d2100 undertaking a simple task
Completing an activity	d2100 undertaking a simple task
Planning and monitoring an activity with correct judgment	b1641 organization and planning b1645 Judgment d2100 undertaking a simple task
Emotional status during an activity	b152 emotional functions
Language skills	b167 mental functions of language

A patient and their therapist will formulate a rehabilitation goal regarding the use of the ATC. This rehabilitation goal will then be used to develop a 5-step GAS. This 5-step GAS will be used to assess changes in reaching the goal in relation to the use of ATC. The GAS score will be evaluated by the therapist and the patient together. When the goal is reached within 5 years, that is, when the patient's goal has reached 0 or above, a new rehabilitation goal will be set.

### Data Management

Every participant will be given a unique number in an unidentified database. The database will be stored in a research computer that will be locked with a secure password, and only the research team will have the password to access the database.

### Data Analysis

#### Linking the Data to the ICF

To answer the first fold of the aim “linking rehabilitation goals regarding the use of ATC to the ICF”, we have developed a preliminary set of ICF categories for persons with neurological diseases in community rehabilitation. We began the development with examining and combining the two ICF core sets for neurological conditions (10, 11), one for the acute phase and the other for the postacute rehabilitation phase and selected the ICF categories that are relevant for persons with neurological diseases in community rehabilitation.

Based on a previous evaluation of rehabilitation goals from the center in this project (unpublished work), we have selected the following ICF categories that are relevant to community rehabilitation. The process of updating this preliminary set of ICF categories will continue throughout the course of the project (Table 3).

**Table 3:** A preliminary set of ICF categories for rehabilitation goals in community rehabilitation for persons with neurological diseases.

**Activity and participation**	**ICF categories**
Chapter 1: learning and applying knowledge	d1551 acquiring complex skills
	d177 making decisions
Chapter 2: general tasks and demands	d210 undertaking a single task
	d2102 undertaking a single task independently
	d2202 undertaking multiple tasks independently
	d2301 managing daily routine
	d2303 managing one's own activity level
	d2400 handling responsibilities
	d2401 handling stress
Chapter 5: self-care	d510 washing oneself
	d5201 caring for teeth
	d570 looking after one's health
Chapter 6: domestic life	d6200 shopping
	d630 preparing meals
	d6402 cleaning living area
	d6403 using household appliances
	d6404 storing daily necessities
	d6405 disposing of garbage
Chapter 8: major life areas	d8501 part-time employment

We will link the rehabilitation goals to the ICF categories according to the linking rules suggested by Cieza et al. (18, 19). The first author and the second author will start with the extraction of meaningful concepts from the goals and the other data. According to the refinement of the linking rules, Cieza et al. (18) suggest that one should ask the question of what the piece of data is about, taking all relevant contextual information into account. Two examples of rehabilitation goals are presented in Table 4. Patient 1 has a diagnosis of ischemic stroke, and patient 2 has a diagnosis of mild brain injury after a traffic accident. We have linked their rehabilitation goals to the corresponding ICF categories.

**Table 4:** Two examples of linking rehabilitation goals to the equivalent ICF categories.

**Rehabilitation goals**	**ICF categories**
**Patient 1**	
Using the time and planning application in his own mobile phone as a reminder, he remembers and knows when to return to his apartment to have lunch at the correct time.	b1442 retrieval and processing of memory b 1642 time management
	d 2301 managing daily routine d570 looking after one's health
	e1151 assistive products and technology for personal use in daily living
**Patient 2**	
Using an electronic calendar to get an overview of daily activities	d 2301 managing daily routine
	e1151 assistive products and technology for personal use in daily living

#### Statistical Analysis

The changes in GAS scores will be used to evaluate the effects of ATC in supporting daily functioning. All patients in the study will have at least one rehabilitation goal in relation to the use of ATC as cognitive support over a 5-year period. Each goal will be rated on a 5-point GAS (between −2 and +2), with the degree of attainment capture for each scale level. The overall GAS will be calculated as described in Turner-Stokes (17). The GAS scores will be incorporated into a single aggregated *T-score* by applying the formula:


$$\begin{array}{l} \text{Overall GAS }=50+\frac{10\sum \left(W_{i}X_{i}\right)}{\sqrt{0.7\sum W_{i}^{2}+{0.3\left(\sum W^{i}\right)}^{2}\;}} \end{array}$$

Where *W*_*i*_ is the weight assigned to a particular goal (if equal weight, *W*_*i*_ = 1), *X*_*i*_=the numerical value achieved (between −2 and +2). The formula will be used to calculate the baseline GAS score and the final GAS score. The difference between the baseline and the final GAS score will be used to indicate effects of the ATC.

Multiple regression analyses will be performed at six different time points to explore the variables that predict goal achievement of the patients. The time points are: 6 months after receiving the ATC, 1 year, 2 years, 3 years, 4 years, 5 years. First, using a standard multiple regression, the following variables were entered in the model: GAS score (between baseline score and final score), number of diagnoses, number of neurological symptoms, living condition, ADL-taxonomy score, Cognitive checklist score and part-time job. The best predictor variable for goal achievement will then identified. Second, hierarchical multiple regression will be performed to assess the ability of this best predictor to predict goal achievement after controlling for the influence of the other variables.

## Conclusion

It is a complex interaction between the person with neurological disease, the activity in which she or he wants to engage in, the environment or context in which the activity occurs, and the ATC available to support the activity. The ICF is a common framework for all health professionals for the coding of functioning. Linking the rehabilitation goals to the ICF categories will provide a comprehensive overview of the goals in relation to the use of cognitive assistive technology and helps to identify the aspects that are lacking in rehabilitation service for persons with neurological diseases. Goal achievement is seldom used to evaluate long-term effects of ATC in persons with neurological diseases and limited attention has been paid to the factors that predict goal achievement in using the ATC as cognitive support in persons with neurological diseases.

## Data Availability Statement

The raw data supporting the conclusions of this article will be made available by the authors, without undue reservation.

## Ethics Statement

The studies involving human participants were reviewed and approved by Swedish Ethical Review Authority. The patients/participants provided their written informed consent to participate in this study.

## Author Contributions

HL led on the writing of this manuscript and NB read and approved the final version.

## Conflict of Interest

The authors declare that the research was conducted in the absence of any commercial or financial relationships that could be construed as a potential conflict of interest.

## Publisher's Note

All claims expressed in this article are solely those of the authors and do not necessarily represent those of their affiliated organizations, or those of the publisher, the editors and the reviewers. Any product that may be evaluated in this article, or claim that may be made by its manufacturer, is not guaranteed or endorsed by the publisher.
